# Enhancing Brain Pregnenolone May Protect Cannabis Intoxication but Should Not Be Considered as an Anti-addiction Therapeutic: Hypothesizing Dopaminergic Blockade and Promoting Anti-Reward

**DOI:** 10.17756/jrds.2015-005

**Published:** 2015-02-27

**Authors:** Kenneth Blum, Marlene Oscar-Berman, Eric R. Braverman, Marcelo Febo, Mona Li, Mark S. Gold

**Affiliations:** 1 Department of Psychiatry & Mcknight Brain Institute, University of Florida College of Medicine, Gainesville, FL, USA; 2 Department of Addiction Research & Therapy, Malibu Beach Recovery Center, Malibu Beach, CA, USA; 3 Department of Psychiatry, University of Vermont, Burlington, VT, USA; 4 Department of Clinical Neurology, Path Foundation NY, NY, USA; 5 Departments of Psychiatry, Neurology, and Anatomy & Neurobiology, Boston University School of Medicine, and Boston VA Healthcare System, Boston, MA, USA; 6 Director of Research, Drug Enforcement Administration (DEA) Educational Foundation, Washington, D.C, USA; 7 Departments of Psychiatry & Behavioral Sciences at the Keck, University of Southern California, School of Medicine, Los Angeles, CA, USA

**Keywords:** Cannabis, CB_1_ type receptors, Pregnenolone, Dopamine, Reward deficiency syndrome (RDS)

## Abstract

Many US states now embrace the medical and recreational use of Cannabis. Changes in the laws have heightened interest and encouraged research into both cannabinoid products and the potential harms of Cannabis use, addiction, and intoxication. Some research into those harms will be reviewed here and misgivings about the use of Pregnenolone, to treat cannabis addiction and intoxication explained. Pregnenolone considered the inactive precursor of all steroid hormones, has recently been shown to protect the brain from Cannabis intoxication. The major active ingredient of *Cannabis sativa* (marijuana), Δ^9^-tetrahydrocannabinol (THC) enhances Pregnenolone synthesis in the brain via stimulation of the type-1 cannabinoid (CB_1_) receptor. This steroid has been shown to inhibit the activity of the CB_1_ receptor thereby reducing many of the effects of THC. While this mechanism seems correct, in our opinion, Vallee et al., incorrectly suggest that blocking CB_1_ receptors could open unforeseen approaches to the treatment of cannabis intoxication and addiction. In this hypothesis, we caution the scientific community that, other CB_1_ receptor blockers, such as, Rimonabant (SR141718) have been pulled off the market in Europe. In addition, CB_1_ receptor blockers were rejected by the FDA due to mood changes including suicide ideation. Blocking CB_1_ receptors would result in reduced neuronal release of Dopamine by disinhibition of GABA signaling. Long-term blockade of cannabinoid receptors could occur with raising Pregnenolone brain levels, may induce a hypodopaminergic state, and lead to aberrant substance and non-substance (behavioral) addictions.

## Introduction

Clinically **S**ubstance Use Disorder (SUD) is a subset of Reward Deficiency Syndrome (RDS) a framework based on a known hypodopaminergic trait (genetic) further impacted by environmental elements (epigenetic) [1]. Most recently the scientific community has been concerned with the potential harms of Cannabis use, addiction and intoxication. Some of those concerns reviewed here, have been amplified because of many US states now embrace medical cannabis and recreational use. Cannabis has been legalized in some states including Colorado, Washington, Alaska and Oregon [2].

### Can we find a solution for cannabis addiction?

In an effort to find a solution to Cannabis abuse and intoxication Vallee et al., suggested that Pregnenolone, maybe useful as an anti-cannabis intoxicant that warrants therapeutic investigation [3]. Pregnenolone is an inactive precursor of all steroid hormones. It is known that the active ingredient of Cannabis Sativa, Δ^9^-tetrahydrocannabinol (THC) enhances the synthesis of Pregnenolone in the brain via stimulation of type-1 cannabinoid (CB_1_) receptor. This pharmacological mechanism provides the scientific rationale for utilizing Pregnenolone to combat Cannabis abuse. However, we provide information that argues this hypothesis.

## Hypothesis

We caution the scientific community that, other CB_1_ receptor blockers, such as Rimonabant (SR141718) have been pulled off the market in Europe and rejected by the FDA due to alarming mood changes including suicide ideation [4]. This enormous societal problem will require more than a singular pharmaceutical solution. Meanwhile, we hypothesize, that blocking dopamine release in the long-term, through the use of, for example, CB_1_ receptor antagonism, is dangerous at least in the long-term.

## New Concerns Regarding Cannabis Use

### Increased potency

The concern about cannabis abuse involves not only traffic violations but unintentional poisoning in toddlers [2]. Moreover, there is growing concern about the use of butane in the development of very potent cannabis concentrated extracts called Budder or Wax among other names. With a consistency similar to that of actual wax or butter, only a small amount of Budder is needed to produce intoxication. Budder is several times more potent than the buds of the cannabis plant that are usually consumed and contains up to 99.7% Δ^9^-tetrahydrocannabinol (THC) and 5%–15% total cannabinoids (CBN/CBD) (5%–25%) [5]. While there may be many reasons for concern related to widespread use of this known intoxicant, we as a society must be concerned with other physiological issues with cannabis abuse. Chronic use of Cannabis involves dopaminergic physiology.

### Developmental implications of maternal cannabis use

An example is that ventral striatal dopamine D2 gene regulation in the offspring is altered by Maternal cannabis use [6]. Based on experimental data DiNieri et al., suggested that epigenetic mechanisms that regulate histone lysine methylation alters developmental regulation of mesolimbic D(2)R in offspring as a result of maternal cannabis use. They further suggested that the ensuing reduction of D(2)R might contribute to addiction vulnerability later in life [6].

Other work from the same group shows that fetal cortical circuitry is disrupted by repeated THC exposure. The endocannabinoid signaling, particularly the temporal dynamics of CB_1_ cannabinoid receptor are rewired in the fetus [7]. Specifically, by interrogating the THC-sensitive neuronal proteome they identified, they found reduced SCG10 mRNA and protein in the hippocampus of cannabis-exposed midgestational human fetuses. These findings suggest that THC induces in utero a rapid degradation in motile axons and microtubule stabilization. Along similar lines Szutorisz et al. determined that parental THC exposure leads to compulsive heroin seeking and altered striatal synaptic plasticity in the subsequent generation(s) [8]. They showed that adolescent exposure to THC results in abnormal heroin seeking behavior in adult F1 offspring. Moreover, parental THC exposure was associated with changes in the mRNA expression of cannabinoid, dopamine, and glutamatergic receptor genes in the striatum. Accordingly, these results could impact offspring phenotype and as such lead to enhanced risk for psychiatric disorders in the subsequent generation.

### Role of epigenetics following cannabis exposure in human adolescents undergoing brain development

Work from Blum’s group reported that perinatal exposure to THC in mice induced supersensitivity in these mice when enkephalins were applied to their vas deferens also suggesting potential risk for opioids in offspring [9]. So,-the question arises; Does marijuana use by teenagers often predate the use of harder drugs? Tomasiewicz et al. reported that regulation of the Pre-Enkephalin (Penk) opioid neuropeptide gene in Nucleus Accumbens Shell (NAcSH) regulates heroin self-administration behavior. Moreover, selective viral-mediated knockdown of Penk in striatopallidal neurons reduces heroin self-administration in adolescent THC-exposed rats, whereas Penk overexpression potentiates heroin self-administration in THC-naïve rats [10].

In addition, adolescent THC exposure mediates Penk up-regulation through reduction of histone H3 lysine 9 (H3K9) methylation in the NAcsh, disrupting the normal developmental pattern of H3K9 methylation [10]. The authors suggest a direct association between THC-induced NAcsh Penk up-regulation and heroin self-administration and that the long-term effects of THC includes the epigenetic dysregulation of Penk. Penk upregulation takes on even more importance in light of the findings of supersensitivity to enkephalin following perinatal exposure to THC [9].

Moreover, adolescence is a critical phase of active brain development and is often characterized by the initiation of marijuana (*Cannabis sativa*) use. Ellgren et al. showed that pre-exposed to THC, this intoxicant induced dynamic changes at various stages of adolescent development in rats. The changes included increased anandamide (brain endogenous cannabis), decreased Met-enkephalin and decreased micro opioid receptors in the Nucleus Accumbens (NAc) [11]. These findings call attention to changes in the mesocorticolimbic endocannabinoid system during adolescence and selective mesocorticolimbic disturbance as a consequence of adolescent cannabis exposure.

In addition, the perinatal administration of marijuana and alcohol in combination or individually resulted in significantly reduced testes weight and plasma testosterone levels in adult male mice [12]. Dalterio et al. also found that in ETOH plus THC-exposed animals, plasma LH was reduced while FSH levels were increased. It is evident from these results that THC and ETOH, either alone or in combination, can affect the development of male reproductive functions in mice [12].

Finally, it is known that high-dose Cannabis use possibly due to enhanced dopamine release in the brain is associated with psychosis, particularly in those vulnerable to psychotic illness. Kuepper et al. [13] observed that THC preferentially increased dopamine release in humans having a high genetic risk for psychosis in both probands with increased risk and in relatives. Indeed, in terms of increased endogenous dopamine release in individuals at risk for psychosis especially at caudate nucleus this is the first study to demonstrate differential sensitivity to Δ^9^-THC [13].

### New Laws and Cannabis abuse?

There is a need to address the problems that have arisen in light of new laws, we caution the scientific community to not embrace long-term utilization of any modality that significantly reduces dopaminergic activation. Our laboratory published on a similar caution when NIDA scientists suggested that raising endogenous brain levels of Kynurenic acid may be a therapeutic agent to treat cannabis dependence [14].

We pointed out that by doing so raising Kynurenic acid will indeed reduce neuronal release of dopamine at mesolimbic sites [15]. Along these lines, we are cognizant that potentially in the short-term the therapeutic approach of blocking the euphoric effects of cannabinoids could be useful in terms of extinction and potential amelioration of THC-induced psychosis intoxication in infants and children. However, this must be avoided in the long-term especially with Pregnenolone.

### Dopaminergic Agonist Modality (DAM)

Clinically, there is enough evidence to caution us against this proposed method of blocking dopamine release by increasing brain Pregnenolone, because it is becoming increasingly important to activate dopaminergic type receptors rather than blocking them or reducing neuronal dopamine release at the NAc [16]. Falenski et al. indirectly supported our hypothesis of the importance of dopamine agonist therapy in the long term, not blockade, by demonstrating control of brain concentration of endogenous anandamide by targeting endocannabinoid catabolic enzymes such as the fatty acid amide hydrolase (FAAH) [17]. Reducing activity of FAAH will in turn increase brain anandamide and enhanced stimulation of CB_1_ receptors ultimately leading to augmented neuronal dopamine release at the NAc and potentially other important brain regions in the reward system.

Volkow’s group showed the profound effects of drugs of abuse like chronic cocaine that induces imbalance between D1 and D2 receptor signaling leading to dopaminergic deficiency [18]. This D1/D2 imbalence has been underscored by the surprising finding of Willuhn et al., showing that when dopamine levels fall in the brain Ventral Medial Striatum (VMS), cocaine self-administration escalates in rats. When they infused the animal with the D2 agonist L-Dopa, the escalation ceased suggesting the importance of a Dopaminergic Agonist Modality DAM [18]. Other work revealed that stressed Wistar-Kyoto, rats that were forced to swim, exhibited an increase in the maximum price that they were willing to pay for cocaine [19]. It was also found that forced swimming in untrained rats results in endogenous opioid deficiency that would lead to attenuated dopamine release in the NAc [20].

### Pregnenolone is not the answer

As we hypothesized earlier, a similar type of a suggestion claiming that blocking dopamine will provide a solution to cannabis abuse. Vallee et al. also deserves cautious attention. They suggest that raising brain Pregnenolone can not only protect the brain from Cannabis intoxication, but be useful as a way to treat Cannabis addiction as well [3].

To reiterate Pregnenolone is considered the inactive precursor of all steroid hormones and it has been recently shown to protect the brain from Cannabis intoxication. Although most steroids are synthesized in the periphery, a number of them are produced in the brain known as neurosteroids. The major active ingredient of *Cannabis Sativa,* Δ^9^-tetrahydrocannabinol (THC) enhances the synthesis of Pregnenolone in the brain via stimulation of type-1 cannabinoid (CB_1_) receptor [3, 21]. It has been shown that this allosteric modulator steroid inhibits the activity of the CB_1_ receptor thereby reducing many of THC effects without altering THC affinity to CB_1_ receptors [3, 22]. Vallee et al. [3] clearly show that Pregnenolone blocks THC-induced neuronal dopamine release in the NAc as well as blocking THC-induced food intake. This group also reported that Pregnenolone reversed self-administration of a CB_1_ agonist WIN55, 212-2 indicating blockade of CB_1_ receptors.

Moreover, it is known that THC induces inhibition of GABA/glutamate release through an interaction with MDMA receptors [23] allowing for “dopamine homeostasis.” These particular THC effects were significantly attenuated by Pregnenolone [3]. In fact, it is suggested that THC-induced production of Pregnenolone exerts a negative feedback on CB_1_ receptor activity so exogenously administered Pregnenolone would result in a heightened inhibitory effect of CB_1_ receptor function. However, in contrast to these effects the sulfated Pregnenolone has been shown by Vallee’s group [3] to increase both dopamine release and the dopaminergic response to morphine in the rat NAc [24]. The dopamine release effect of the sulfated Pregnenolone seems to be incongruent and unresolved relative to their current hypotheses.

## Conclusion

With these unanswered questions about the actual effects of Pregnenolone and the anti-reward risks for mood changes including suicide ideation further research is required. However, the search for an antidote for the new danger of cannabis intoxication of toddlers and children and warnings against cannabis use during gestation, nursing and adolescence should be encouraged in the medical and scientific community. It may be prudent to consider dopamine agonists rather than antagonistic therapy for long term maintenance as observed for example, now in many clinical trials utilizing a putative D2 agonist such as KB220Z [25, 26].
